# Socioeconomic differences in purchases of more vs. less healthy foods and beverages: Analysis of over 25,000 British households in 2010^☆^

**DOI:** 10.1016/j.socscimed.2013.05.012

**Published:** 2013-09

**Authors:** Rachel Pechey, Susan A. Jebb, Michael P. Kelly, Eva Almiron-Roig, Susana Conde, Ryota Nakamura, Ian Shemilt, Marc Suhrcke, Theresa M. Marteau

**Affiliations:** a Behaviour and Health Research Unit, Institute of Public Health, University of Cambridge, Cambridge CB2 0SR, UK; b MRC Human Nutrition Research, Elsie Widdowson Laboratory, Cambridge, UK; c Public Health Excellence Centre, National Institute for Health and Clinical Excellence, London, UK; d School of Medicine, Health Policy and Practice, University of East Anglia, Norwich, UK

**Keywords:** Britain, Socioeconomic status, Diet, Purchasing, Scanner data, Attitudes, Health inequalities

## Abstract

Socioeconomic inequalities in diet-related health outcomes are well-recognised, but are not fully explained by observational studies of consumption. We provide a novel analysis to identify purchasing patterns more precisely, based on data for take-home food and beverage purchases from 25,674 British households in 2010. To examine socioeconomic differences (measured by occupation), we conducted regression analyses on the proportion of energy purchased from (a) each of 43 food or beverage categories and (b) major nutrients. Results showed numerous small category-level socioeconomic differences. Aggregation of the categories showed lower SES groups generally purchased a greater proportion of energy from less healthy foods and beverages than those in higher SES groups (65% and 60%, respectively), while higher SES groups purchased a greater proportion of energy from healthier food and beverages (28% vs. 24%). At the nutrient-level, socioeconomic differences were less marked, although higher SES was associated with purchasing greater proportions of fibre, protein and total sugars, and smaller proportions of sodium. The observed pattern of purchasing across SES groups contributes to the explanation of observed health differences between groups and highlights targets for interventions to reduce health inequalities.

## Highlights

• We give a novel, detailed account of purchasing by SES in 25,000 British households.
• Lower SES groups purchased a greater proportion of less healthy food and drink.
• Higher SES groups purchased proportionally more high-fat dairy and alcohol.
• Overall, SES differences were less marked for nutrients than for food groups.
• We highlight food groups to target for interventions to reduce health inequalities.

## Introduction

Recent focus in the UK and elsewhere on the social determinants of health inequalities (Commission on Social Determinants of Health, 2008; Marmot, 2010) has raised the question of how social, economic and political environments influence health outcomes such as obesity and heart disease at the individual- and population-levels (Galea, Riddle & Kaplan, 2010; Kelly, 2010a). The literature suggests that the pathways by which environments influence behaviour, diet and ultimately health will be complex (Galea et al., 2010; Taylor, Repetti & Seeman, 1997; Warnecke et al., 2008), that there will be different levels of explanations socially and individually (Galea et al., 2010; Hawe, Shiell & Riley, 2009; Kelly, 2010b), and that there is a need for well-conducted empirical studies (Östlin et al., 2011).

Food and drink purchasing are determinants of consumption, yet their role in the aetiology of health disparities is underexplored. While it is often argued that there are social class-based patterns in dietary behaviours, beyond social disparities in the consumption of fruits and vegetables (De Irala-Estévez et al., 2000; Diez-Roux et al., 1999; Galobardes, Morabia, & Bernstein, 2001; Giskes, Avendaňo, Brug, & Kunst, 2010), relatively little evidence has accumulated to support this claim from representative surveys of food consumption. Moreover, such studies are vulnerable to bias due to misreporting and measurement error (Carriquiry, 2003; Poslusna, Ruprich, de Vries, Jakubikova & van't Veer, 2009; Rennie, Coward & Jebb, 2007). The small corpus of studies that has explored purchasing replicate the finding that fruit and vegetable purchasing is socially patterned, as well as suggesting that lower SES is associated with purchasing cheaper, less nutrient-rich calories, but the analyses tend to be limited in terms of scale and/or precision (e.g. using very broad outcome measures) (Appelhans et al., 2012; French, Wall & Mitchell, 2010; Turrell et al., 2009; Turrell, Hewitt, Patterson, Oldenburg & Gould, 2002; Turrell & Kavanagh, 2006; UK Department for Environment, 2011; Vinkeles Melchers, Colagiuri & Gomez, 2009). A more precise description of social patterning of food and drink purchasing, relating the types of foods purchased to their nutritional content, would facilitate assessment of the potential for any observed behavioural differences to contribute to disparities in health outcomes and identify potential targets for intervention.

The analysis reported in this paper contributes to this objective by addressing two core questions:

1. How do patterns of purchasing of (a) food and drink categories, and (b) the nutritional content of food and drink, vary by SES?
2. Are any observed purchasing patterns consistent with the extant empirical evidence for differences in health outcomes by SES?

## Methods

### Kantar WorldPanel (KWP) dataset

KWP's commercial panel comprises over 25,000 British households, recruited via stratified sampling, with targets set for region, household size, age of main shopper and occupational group. KWP offer vouchers from high street retailers as compensation for participation. Households provide demographic information when joining the panel, followed by annual updates. Households record all purchases (from all store types) brought back into the home using barcode scanners (with barcodes provided to record non-barcoded products like fruit). KWP match scanned records to their nutritional data.

To be included in KWP's final datasets, households must meet quality control criteria (meeting thresholds for data recording and purchasing volume/spend (based on household size) every four weeks). Panellists also upload digital images of checkout receipts, which KWP use to verify the accuracy of scanner data. We obtained KWP data on take-home purchasing of food and drink for the 52 weeks ending 26th December 2010, and analysed all households that reported at least 12 weeks' data (*n* = 25,674; see Table 1 for sample characteristics).

**Table 1:** Sample characteristics.

Demographic		N (%)
Main shopper age group	18–29	2729 (11%)
	30–39	5680 (22%)
	40–49	5363 (21%)
	50–59	4602 (18%)
	60–69	3944 (15%)
	70+	3356 (13%)
Main shopper gender	Female	20180 (79%)
Main shopper ethnicity	White	23597 (95%)
	Non-white	1187 (5%)
Occupational group (SES)	A & B: Higher Managerial and Professional	5469 (21%)
	C1 & C2: White Collar and Skilled Manual	14066 (55%)
	D & E: Semi-skilled and Unskilled Manual	6139 (24%)
Number of adults in household	1	5861 (23%)
	2	14783 (58%)
	3	3172 (12%)
	4+	1858 (7%)
Number of children in household	0	16419 (64%)
	1	3934 (15%)
	2	3798 (15%)
	3	1168 (5%)
	4+	355 (1%)
Region	London	4511 (18%)
	Midlands	4296 (17%)
	North East	1356 (5%)
	Yorkshire	2683 (10%)
	Lancashire	3077 (12%)
	South	2528 (10%)
	Scotland	2211 (9%)
	Anglia	2091 (8%)
	Wales and West	2048 (8%)
	South West	873 (3%)
Total number of households		25,674

### Key variables

#### Socioeconomic position

Socioeconomic position is based on head-of-household occupation (based on the UK Registrar Generals' classification), comprising three groups: Higher Managerial and Professional (A&B); White Collar and Skilled Manual (C1&C2); and Semi-skilled and Unskilled Manual (D&E). Notwithstanding its well-known limitations (Graham & Kelly, 2004), this classification has been used to capture differences in population patterns of health over several decades.

#### Food/beverage categories

We classified all scanned food and beverage products into one of 43 categories (based on an established food group classification scheme (Johnson, Mander, Jones, Emmett & Jebb, 2008) and considering products' nutritional characteristics: see supplement). This process began with individual products, allowing for sharper delineation of categories than in most previous work. Of these categories, 22 were paired in the sense that they represented healthier and less healthy versions of the same food or beverage (e.g., high-fibre and lower-fibre bread).

We then classed each product category as ‘healthier’, ‘neutral’ (i.e. neither particularly healthy nor unhealthy) or ‘less healthy’ (see Table 2 for category groupings). Within the paired categories, the healthier category was classed as ‘healthier’, and its counterpart as ‘less healthy’. The remaining categories were distributed according to their nutritional characteristics (see supplement). Of the 43 categories, 21 represent ‘less healthy’, seven ‘neutral’ and 15 ‘healthier’ foods or beverages.

**Table 2:** Differences by SES group in the mean percentage of energy purchased from each food/beverage category.

		Mean percentage of energy from category (all SES groups)	Regression analyses (reference group: C1&C2)					
			A&B			D&E		
			Coefficient (exponentiated)	Standard Error	Significance	Coefficient (exponentiated)	Standard Error	Significance
Less healthy	**Sweet snacks/puddings**	10.15%	0.93	0.019		1.14	0.017	***
	**Margarines/cooking oils**	7.33%	0.88	0.031	*	1.08	0.030	
	**Low-fibre bread products**	7.03%	0.88	0.019	***	1.08	0.018	*
	Processed meats	5.02%	0.90	0.025		1.01	0.022	
	Savoury snacks	4.67%	0.95	0.027		1.01	0.029	
	**Chocolate/confectionery**	4.66%	0.98	0.025		1.14	0.022	***
	**High-fat cheese**	3.53%	1.12	0.024	**	0.87	0.025	***
	**Butter/animal fats**	3.14%	1.70	0.056	***	0.64	0.059	***
	**Regular pasta/rice**	2.97%	1.22	0.029	***	0.82	0.032	***
	High-energy drinks	2.32%	0.94	0.028		1.04	0.029	
	**Processed potato**	2.14%	0.67	0.044	***	1.37	0.041	***
	Low-fibre cereals	2.08%	1.00	0.047		0.99	0.046	
	High-fat milk	1.66%	0.92	0.054		1.09	0.053	
	Less healthy ready meals	1.12%	0.89	0.041		1.03	0.038	
	**Wine**	1.07%	2.03	0.050	***	0.34	0.050	***
	**Beer and cider**	1.03%	1.13	0.051		0.55	0.052	***
	**Spirits**	0.90%	1.22	0.053		0.70	0.051	***
	**High-fat dairy (ex. cheese)**	0.71%	1.42	0.044	***	0.72	0.046	***
	**High-energy soups**	0.35%	1.12	0.036		0.81	0.035	***
	**High-energy sauces**	0.23%	1.18	0.039	*	0.65	0.038	***
	Flavoured alcoholic beverages	0.11%	0.89	0.034		1.12	0.035	
Neutral	Carcass meats/poultry	4.11%	0.95	0.031		0.95	0.027	
	**Morning goods**	3.39%	1.06	0.026		0.85	0.027	***
	**Other lean protein**	2.76%	1.11	0.021	***	0.92	0.021	
	**Canned/dried fruit**	0.63%	1.49	0.041	***	0.72	0.040	***
	**Spreads/condiments**	0.39%	1.44	0.036	***	0.71	0.035	***
	Dairy drinks	0.10%	0.90	0.037		1.12	0.035	
	Non-alcoholic beer	0.00%	1.04	0.012		0.97	0.009	
Healthier	**Low-fat milk**	3.93%	1.19	0.035	***	0.77	0.038	***
	**Low-fat dairy (ex. cheese)**	3.52%	1.18	0.023	***	0.87	0.026	***
	**High-fibre cereals**	3.43%	1.41	0.040	***	0.73	0.040	***
	**Fresh fruit**	3.16%	1.27	0.023	***	0.69	0.028	***
	Potatoes	2.95%	1.04	0.031		0.88	0.033	
	**High-fibre bread products**	2.07%	1.42	0.040	***	0.68	0.042	***
	**Vegetables**	1.87%	1.16	0.016	***	0.84	0.018	***
	Legumes	1.53%	1.03	0.023		0.92	0.024	
	Healthier ready meals	1.18%	0.89	0.040		0.91	0.039	
	**Juice**	1.05%	1.81	0.043	***	0.56	0.047	***
	Low-energy sauces	0.78%	1.00	0.026		0.91	0.026	
	**Low-fat cheese**	0.38%	1.23	0.038	***	0.78	0.039	***
	Low-energy drinks	0.26%	0.94	0.024		1.06	0.023	
	**Brown pasta/rice**	0.19%	1.47	0.045	***	0.70	0.035	***
	**Low-energy soups**	0.05%	1.04	0.034		0.79	0.031	***

*p < 0.05; **p < 0.01; ***p < 0.001 (adjusted using Bonferroni's correction).

Based on multiple regressions with age, gender, ethnicity, no of adults in household, no of children in household, region; Categories with significant differences marked in bold.

### Data aggregation and analysis

For each household, the proportions of total energy purchased from each category and each major nutrient were calculated. Data for fibre and sodium were expressed as grams per 1000 kcal.

Multiple regression analyses (using Stata MP version 11, ‘regress’ command, with bootstrapped standard errors and logged outcome variables due to skewed data, including many zero purchasers) were conducted for each category and nutrient separately, with dummy variables for SES group, and controlling for age, gender, ethnicity (white; non-white), number of adults in the household, number of children in the household, and region. Reported significance levels were adjusted for multiple testing using Bonferroni's correction.

## Results

### Category-level

Table 2 shows the mean percentage of energy purchased from each food/beverage category. For 28 of the 43 categories we observed statistically significant differences between SES groups A&B or D&E and reference group C1&C2. These were generally small in terms of absolute percentages (the largest differences identified here were in the region of 1–2 percentage points overall).

Lower SES groups bought greater proportions of their total energy from 9/21 less healthy categories (including sweet snacks and puddings, processed potatoes and low-fibre bread products), accounting for 34.8% of group D&E's total energy purchased (6.5 percentage points more than group A&B). In contrast, higher SES groups bought greater proportions of energy from 5/21 less healthy categories (including wine, high-fat cheese and high-fat dairy), accounting for 15.3% of group A&B's total energy purchased (2.7 percentage points more than group D&E).

A greater proportion of energy was bought from 4/7 neutral categories by higher SES groups (together accounting for 7.7% of group A&B's total energy, compared to 6.7% for group D&E), whereas no neutral categories were purchased proportionally more by lower SES groups.

For healthier categories, there was a difference by SES group for 10/15 categories, with all these due to a higher proportion of energy being bought by higher SES groups. For example, in the juice category, on average the percentage of energy purchased from juice was 81% greater in the A&B group than in C1&C2 group, while percentage of energy from juice purchased by group D&E was 44% less than group C1&C2. Together these accounted for 21.8% of group A&B's total energy purchased, an extra 4.2 percentage points compared to group D&E.

**Fig. 1:**
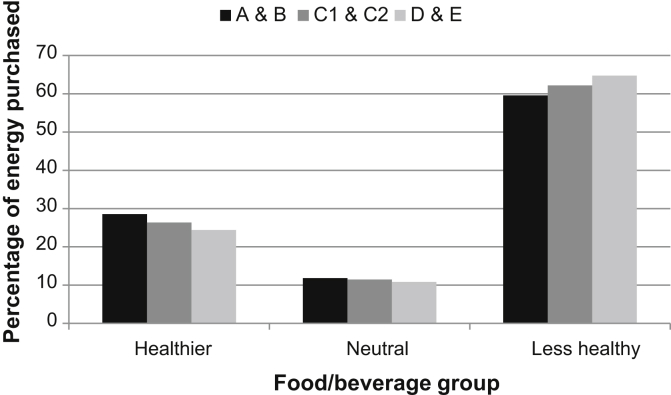
Percentage of energy purchased from healthier, neutral and less healthy categories by each SES group. N.B. All the SES group differences depicted are significant at *p* < 0.001.

Fig. 1 shows the percentage of energy purchased from aggregated less healthy, neutral and healthier category groupings by SES group. The percentage of energy purchased from the less healthy grouping was 60%, 62% and 65%, and from the healthier grouping 28%, 26% and 24%,for groups A&B, C1&C2, and D&E, respectively. Using multiple regression, groups A&B and D&E were significantly different from group C1&C2 for all three category groupings (all at *p* < 0.001).

### Nutrient-level

Table 3 shows purchasing of nutrients by SES. Higher SES groups purchased more grams of fibre per 1000 kcal than lower SES groups, as well as a greater percentage of their energy from total sugars and protein. In addition, group A&B purchased 3% less sodium per 1000 kcal than group C1&C2.

**Table 3:** Differences by SES group in the percentage of total energy from total fat, saturated fat, carbohydrates, sugars and protein, and grams per 1000 kcal of sodium and fibre.

		Mean	Regression analyses (reference group: C1&C2)					
			A&B			D&E		
			EXP(B)	SE	Sig	EXP(B)	SE	Sig
Percentage of total energy	Total fat	42.39%	1.00	0.002		1.01	0.002	
	Saturated fat	12.80%	1.01	0.004		1.00	0.004	
	Carbohydrates	44.49%	1.00	0.002		1.01	0.002	
	Total sugars	13.94%	1.03	0.005	***	0.98	0.005	*
	Protein	13.12%	1.02	0.004	*	0.96	0.003	***
Grams per 1000 kcal	Sodium	1.50 g	0.97	0.007	**	1.03	0.006	
	Fibre	9.11 g	1.05	0.005	***	0.94	0.005	***

*p < 0.05; **p < 0.01; ***p < 0.001 (adjusted using Bonferroni's correction).

Based on multiple regressions with age, gender, ethnicity, no of adults in household, no of children in household, region.

## Discussion

Household purchasing differed by SES group for 28 of our 43 categories. The extent of the differences identified here are more widespread than suggested by previous work, with the proportions purchased from dairy products, pasta and rice, bread products, processed potatoes, sweet snacks and puddings, and wine all showing significant differences by SES. In general, purchasing by lower SES groups was characterised by proportionally less energy from healthier food categories (4 percentage points difference between groups A&B and D&E) and proportionally more energy from less healthy food categories (5 percentage points difference between groups A&B and D&E). While the SES differences were mainly small in absolute terms, at the population-level and over time, these differences are likely to have cumulative effects on health risks.

Given that health considerations played a role in determining the categories used in this study, category-level differences would be expected to be reflected at the nutrient-level. Differences were indeed observed, with proportionally more purchasing of fibre, protein and total sugars by the higher SES groups, and proportionally less sodium purchased by the highest SES group. Both sodium and fibre have been linked to health outcomes, with excess sodium consumption being associated with high blood pressure and elevated risk of stroke (Intersalt Cooperative Research Group, 1988) and higher fibre intake potentially playing a role in preventing obesity (Slavin, 2005). These results suggest that the shopping baskets of higher SES groups are healthier in these regards than those of lower SES groups, which may be a contributing factor to observed health inequalities.

However, the other differences in nutrient purchasing are less relevant to health outcomes. Health effects of total protein intake are equivocal (Gilbert, Bendsen, Tremblay & Astrup, 2011), and the role of total sugars may be confounded by differences in the sources of the sugar, e.g. fruit, dairy or added sugars (Murphy & Johnson, 2003). Whilst saturated fat consumption has been a key concern for health, no socioeconomic differences were found in the proportion of saturated fat purchased. However, when the latter result is considered in conjunction with category-level data, it seems likely that higher SES households were obtaining more of their saturated fats from dairy, which may contain more beneficial nutrients compared to many other sources of saturated fat (German et al., 2009). Such comparisons highlight and reinforce the value in considering broader aspects of diet than nutrients alone in developing interventions to tackle obesity and other diet-related diseases.

The current study has a number of strengths. It has extended previous work on socioeconomic differences in food purchasing, using a large dataset based on observed data and narrower health-based categories than in previous studies, to reveal small socioeconomic differences in purchasing across a wider range of categories. It used a large sample that may inform the design, tailoring and targeting of population interventions (Kelly, 2010a) that aim both to reduce SES differences in purchasing and to increase the healthiness of food purchasing overall (Capewell & Graham, 2010).

It also has a few limitations: as the data do not include out-of-home purchasing, the socioeconomic differences reported need to be treated with caution as they represent only part of the diet. Higher SES groups generally consume more out-of-home than lower SES groups (Lachat et al., 2012), and the types of foods consumed out-of-home by lower SES groups may be less healthy than those consumed by higher SES groups (French et al., 2010; Thornton, Crawford & Ball, 2011). The current study explicitly focused on purchasing rather than consumption behaviour, so food waste and dietary intake were not assessed. Regarding the representativeness of the dataset, the overall low volumes of food and beverages recorded suggest underreporting. However, KWP supply weighting variables to adjust for such underreporting and similar results (available on request) were found when we conducted a sensitivity using these weighted data.

The UK government's ‘Healthy Lives, Healthy People’ White Paper highlights the importance of ‘improving the health of the poorest, fastest’ (UK Government, 2010, 4). The current study highlights particular product categories that may be contributing most to inequalities in diet: low-fibre bread products, sweet snacks and puddings, and confectionery between them contributed 24% of group D&E's energy, compared to 20% for group A&B. At the nutrient-level, saturated fat and salt have been the primary focus of health campaigns (given their associations with higher blood pressure and increased risk of cardiovascular disease (He, Li, & MacGregor, 2013; Hooper et al., 2012)), yet we find only limited evidence of differences in purchasing of these nutrients. In contrast, fibre, which shows the most striking findings with regard to socioeconomic differences in purchasing in the current study, has received less consideration. Further work is needed to understand the socioeconomic patterns of food purchasing observed in this study; for example, price, availability and preferences may help explain differences. While the results here do not directly address policy interventions, they do reveal potential targets to reduce inequalities in household food purchasing, which have received less attention to date than interventions focused on individual-level consumption.
