# Supplementary material for: Socioeconomic differences in purchases of more vs. less healthy foods and beverages: Analysis of over 25,000 British households in 2010
Source: Soc Sci Med. 2013 Sep;92:22–6. doi: 10.1016/j.socscimed.2013.05.012 (PMC3726935; doi:10.1016/j.socscimed.2013.05.012)
Supplement: Supplementary file 1 [file mmc1.docx]

**Supplementary material: Food/beverage categories**

*Categorisation of food/beverage products*

All food and beverage products in the dataset were divided into one of 43 categories (based on previous food group classifications ([Johnson et al., 2008](#_ENREF_17)) and definitions for energy- and nutrient-based classifications from European legislation (Regulation (EC) No 1924/2006 for nutrition and health claims) and the Department of Health (UK).

22 categories were paired in the sense that they represented healthier and less healthy versions of the same type of food or beverage. The categories are listed below, subdivided into paired and non-paired categories:

**FOODS**

**Paired categories:**

*Bread products (incl. savoury pancakes, Yorkshire pudding, pastry, naan bread, savoury crackers, pizza bases)*

1. High-fibre bread products (where high fibre = at least 6 g of fibre/100 g OR at least 3 g fibre/100 kcal)

2. Lower-fibre bread products (incl. pizza bases)

*Pasta, rice, and other grains*

3. Non-wholemeal pasta, rice, noodles, couscous, gnocchi, other grains

4. Wholemeal pasta, rice, noodles, couscous, gnocchi, other grains

5. Potatoes

6. Processed potato products (e.g., chips, pre-prepared roast potatoes, instant mash)

*Cheese*

7. High-fat cheese (more than 20g fat /100g)

8. Lower-fat cheese

*Other dairy*

9. High-fat other dairy (incl. yoghurt, ice cream, cream) (more than 20g fat /100g)

10. Lower-fat other dairy (incl. yoghurt, ice cream, cream)

*Sauces*

11. Lower-energy density sauces (ED <1.5 kcal/g or ml, probably mainly tomato-based)

12. Higher-energy density sauces (ED 1.5+ kcal/g or ml, probably mainly dairy- or oil-based)

*Soups*

13. Lower-energy density soups (less than 40kcal/100g)

14. Higher-energy density soups (more than 40kcal per 100g)

*Ready meals (incl. pizza, pies, lasagne, curry, quiche, tinned meals (e.g. sausage and beans), pot noodle, pasta in sauce etc.)*

15. ‘Healthier’ ready meals (FSA score of -1 or less)

16. ‘Less healthy’ ready meals (FSA score of 0 or more)

*Breakfast cereals*

17. High-fibre breakfast cereals (where high fibre = at least 6 g of fibre/100 g OR at least 3 g fibre/100 kcal)

18. Lower-fibre breakfast cereals

**Non-paired categories**

*Morning goods*

19. Morning goods (croissants, muffins, crumpets)

*Fruits, vegetables, and legumes*

20. Fresh fruit

21. Canned, stewed, baked or dried fruit

22. Vegetables, excluding legumes and potatoes

23. Legumes (incl. baked beans), seeds and unprocessed nuts

*Meat, meat substitutes and eggs*

24. Processed meat (incl. mince, meatballs, bacon, ham, luncheon meats, corned beef, pate, burgers, sausages)

25. Carcass meats and poultry (lean red meats and poultry (incl. battered/breadcrumbed))

26. Other lean protein (fresh fish and shellfish, eggs, quorn, other meat substitutes)

*Fats and oils*

27. Butter and animal fats

28. Margarine and vegetable oils

*Savoury spreads and condiments*

29. Savoury spreads and condiments (savoury spreads, dips, condiments, table salt, stock cubes, herbs and spices)

*Snacks and treats*

30. Chocolate and confectionery (incl. chocolate spread, jelly, jam, honey)

31. Savoury snacks (incl. crisps and processed nuts, peanut butter, olives)

32. Sweet snacks and puddings (incl. cakes, pastries, sweet biscuits, sweet home cooking and cake-based puddings)

**BEVERAGES**

**Paired categories**

*Milk*

33. Reduced-fat milk (semi-skimmed (1.8g of fat per 100ml) or lower fat)

34. Higher-fat milk

*Soft drinks (excl. dairy-based drinks and juice; incl. carbonated drinks, squashes/cordials, hot/powdered drinks, water)*

35. Sugar-sweetened drinks

36. Low-energy drinks (no more than 20kcal/100ml)

**Non-paired categories**

*Dairy-based drinks (excl. milk; must contain at least 3.5g of protein per 100ml)*

37. Flavoured milks and yoghurt drinks

*Juice*

38. Fruit juice (100%)

*Alcohol*

39. Standard beer, cider

40. Standard wine

41. Spirits (incl. fortified wine)

42. Low-/no-alcohol beer, cider, wine

43. Flavoured alcoholic beverages (e.g., alcopops, shandy)

*Classification of food/beverage categories*

Each category was then classed as ‘healthier’, ‘neutral’ or ‘less healthy’. Within the 22 paired categories, the healthier option was automatically classed as healthier, and its counterpart as less healthy. The remaining categories were distributed according to their score from the FSA Nutrient Profiling model ([Rayner et al., 2005](#_ENREF_26)):

- Categories with a mean score using the of ≥5 were classed as less healthy
- Categories with a mean score of ≤-3 were classed as healthier
- Categories with a mean score of -2 –4 were classed as neutral

(See Table S1 for the mean FSA score for each of the 43 categories.)

Alcoholic drinks are not included in this scoring scheme and all alcohol categories were classed as less healthy.

**Table S1: Mean FSA score by food/beverage category**

| **Category** | **Mean FSA Score** | **Standard deviation** |
| --- | --- | --- |
| Vegetables | -7.68 | 2.21 |
| Potato | -6.89 | 0.61 |
| Legumes | -5.42 | 6.22 |
| Fresh fruit | -4.87 | 1.93 |
| Brown pasta/ rice | -3.48 | 2.65 |
| Juice | -3.17 | 1.26 |
| Light ready meals | -2.69 | 2.52 |
| High fibre bread products | -1.58 | 6.65 |
| Regular pasta/ rice | -0.55 | 4.06 |
| Low fat milk | -0.27 | 0.75 |
| Processed potato | -0.21 | 2.86 |
| Regular ready meals | -0.18 | 4.47 |
| Low energy drinks | -0.13 | 0.77 |
| Wine | 0.00 | 0.07 |
| Non-alcoholic beer | 0.00 | 0.00 |
| Beer and cider | 0.01 | 0.21 |
| Other lean protein | 0.64 | 4.78 |
| High fat milk | 0.68 | 1.36 |
| Carcass meats/ poultry | 0.70 | 5.07 |
| Low energy soups | 0.94 | 1.92 |
| High fibre cereals | 1.10 | 5.84 |
| Morning goods | 1.12 | 5.40 |
| Dairy drinks | 1.13 | 2.11 |
| Spirits | 2.13 | 1.60 |
| High energy soups | 2.27 | 5.74 |
| High energy drinks | 2.58 | 6.83 |
| FABs | 2.95 | 1.23 |
| Spreads/ condiments | 3.37 | 5.17 |
| Canned/ dried fruit | 3.64 | 7.21 |
| Low fat dairy (ex. cheese) | 4.47 | 5.82 |
| Low fibre bread products | 5.85 | 7.82 |
| Low energy sauces | 6.05 | 5.27 |
| Low fibre cereals | 7.14 | 5.18 |
| Savoury snacks | 8.22 | 6.89 |
| Low fat cheese | 10.20 | 8.59 |
| Processed meats | 10.76 | 7.61 |
| Sweet snacks/ puddings | 10.99 | 7.46 |
| High energy sauces | 11.47 | 6.67 |
| High fat dairy (ex. cheese) | 15.35 | 3.48 |
| Chocolate/ confectionery | 17.07 | 8.08 |
| High fat cheese | 18.61 | 4.69 |
| Margarines/ cooking oils | 19.36 | 3.42 |
| Butter / animal fats | 21.95 | 4.39 |
